# Α molecular epidemiological analysis of adenoviruses from excess conjunctivitis cases

**DOI:** 10.1186/s12886-017-0447-x

**Published:** 2017-04-24

**Authors:** A. Balasopoulou, P. Κokkinos, D. Pagoulatos, P. Plotas, O. E. Makri, C. D. Georgakopoulos, A. Vantarakis

**Affiliations:** 1 Environmental Microbiology Unit, Department of Public Health, Medical School, University of Patras, Patras, Greece; 2 Department of Ophthalmology, Medical School, University of Patras, Patras, Greece

**Keywords:** Adenovirus, Conjunctivitis, Eye, Infection, Molecular detection

## Abstract

**Background:**

Τo perform a molecular epidemiological analysis of viral conjunctivitis among excess conjunctivitis cases recorded at the University Hospital of Patras, Greece, for the period March to June 2012.

**Methods:**

A structured questionnaire containing demographic and clinical data was developed in order to collect retrospective data on the cases. Eye swab specimens were collected and molecular detection of adenoviruses was performed by nested PCR. Positive results were confirmed by sequencing. To determine the relatedness between the isolated sequences, a phylogenetic analysis was conducted.

**Results:**

The epidemiological analysis (including retrospective data) included 231 conjunctivitis cases (47.1% male, and 52.8% female). Based on clinical features 205 of the cases were diagnosed of viral origin (46.3% male and 53.7% female), 4 of bacterial origin (50% male and 50% female) while 22 were undefined conjunctivitis. The outbreak excess cases (included 156 cases) affected all age groups regardless gender predilection. For the positive samples indicated that 29 samples (72.5%) were AdV17, and 5 (12.5%) as AdV54.

**Conclusions:**

Molecular analysis could define the cause of viral conjunctivitis, while epidemiological data contributed to the assessment of the risk factors and underlined possible preventive measures. This study is one of the very few on viral conjunctivitis in Greece. This outbreak underscores the need for a national surveillance system for acute infectious conjunctivitis outbreaks. The epidemiological as well as molecular investigation on HAdV ocular infections is rather absent in Greece, which has no surveillance system for viral conjunctivitis.

## Background

Conjunctivitis is one of the most frequent ocular disorders observed in clinical practice [1]. Conjunctival infections are caused both in sporadic and epidemic form, due to a variety of microorganisms, including bacteria [2], viruses [3] and parasites [4]. The leading cause of acute viral conjunctivitis is human adenoviruses (HAdVs) [5, 6]. About 15–70% of all conjunctivitis cases worldwide are associated with HAdVs [1] where clinical manifestations include epidemic conjunctivitis (EC), pharyngoconjunctival fever and non-specific follicular conjunctivitis [5, 7]. Although most adenoviral infections have been described as mild and self-limited, HAdVs have been associated with severe infections in both immunocompromised and healthy individuals [8]. HAdVs cause outbreaks in a wide range of settings, such as military recruits [5, 9, 10] or hospitals [11]. Most EC outbreaks are community-based and transmitted from person to person, by contact with respiratory or ocular secretions, by fingers or through contaminated ophthalmologic instruments. In fact, most of the described epidemic outbreaks represent infections of a common source, which may include inadequately chlorinated swimming pools or contaminated ophthalmology unit.

The HAdVs belong in the genus of Mastadenovirus of the family of Adenoviridae consisting of more than 60 types [12], grouped into seven species (A to G) based on the serological, biochemical and genetic properties [13, 14].

The detection of adenovirus in conjunctival swabs is notifiable to the local health departments in Germany. The German national surveillance system captured an outbreak with 1024 cases from January to April 2004. Meanwhile, the German Armed Forces experienced an outbreak of conjunctivitis affecting 6378 soldiers [15]. In 2010 (as of 13 October 2010,), the number of adenovirus conjunctivitis cases reported to the Robert Koch Institute in Berlin, Germany, has increased by more than 250% compared with same period in the previous 2 years [16]. The epidemiological as well as molecular investigation on HAdV ocular infections is rather absent in Greece, which has no surveillance system for viral conjunctivitis. The impact of HAdV infection is unknown, and very few epidemiological studies on adenoviral conjunctivitis have been reported so far. Furthermore, there is no previous molecular epidemiological study of adenoviral conjunctivitis in Greece. The present study refers to an epidemiological and molecular investigation of conjunctivitis excess cases and aims to identify the predominant types. The purposes of this study are to enrich the very poor data of epidemiological molecular studies on viral conjunctivitis in Greece, to demonstrate the benefit of clinical surveillance as a tool to determine the epidemiology of viruses circulating in each community, and to underline the need for the design and support of similar long-term studies in our country.

## Methods

### Epidemiological investigation

Patras is Greece’s third largest urban area and the regional capital of West Greece, located in northern Peloponnese. It has 177.245 inhabitants per the 2011 census [17], and a typical Mediterranean climate.

In the first days of March 2012, reports of conjunctivitis cases to the University Hospital of Patras (UHP), increased markedly. All conjunctivitis cases referred to the UHP during the period from January 1, 2012 to July 31, 2012 (weeks 1–30) were evaluated retrospectively using medical records of the hospital. A structured questionnaire was developed and used to gather demographic and clinical data from in- and out-patients referred to the UHP, in the period between February 27, 2012 to June 17, 2012 (weeks 9–24). The questionnaire included information about gender, age, symptoms, date of onset of symptoms. Questionnaires were completed by the physicians examined the patients. All the patients gave informed consent to participate in the research study. To establish the retrospective data for the epidemiological investigation, conjunctivitis cases either hospitalized or treated in the Outpatients Department of the UHP, from January till July 2011, were evaluated with a structured questionnaire containing data on age, sex, symptoms, connection with other case, travel abroad, participation in a setting (e.g. school, etc.). Also, the environmental data (temperature, relative humidity, pressure, precipitation, wind speed) from 2011 and 2012 were collected by the Laboratory of Atmosphere Physics.

An outbreak case was defined as any clinically suspected case of adenoviral conjunctivitis in the hospital during the period from February 27 to June 17, 2012. More precisely the diagnosis of adenoviral conjunctivitis is set clinically and is based on its characteristic clinical features like eyelid oedema, watery discharge, itchy eyes, conjunctival hyperemia and palpebral conjunctival follicles, subconjunctival hemorrhages, pseudomembranes, punctuate keratopathy, subepithelial corneal infiltrates and ipsilateral tender palpable pre-auricular lymph node.

### Molecular analysis

Forty- eight (48) conjunctival swabs were collected from patients referred for acute conjunctivitis to the UHP during the period of the excess cases (weeks 9–24). The swabs were transferred into a test tube containing 1 ml of Eagle’s MEM with 0.5% bovine serum albumin [18] and were used as a clinical sample for a later laboratory diagnosis. The samples were transferred to the laboratory into a cool box and they were immediately subjected to virological analysis. Viral nucleic acids were extracted from concentrated samples using the QIAamp viral RNA mini kit (Qiagen), according to the manufacturer’s protocol and stored in aliquots at −70 °C. Nested PCR techniques have been used for the detection of HAdVs, according to previously published protocols [19–22]. Extracted DNA was amplified by a two-step nested polymerase chain reaction (PCR) using specific primers for a conserved region of hexon gene, that yielded a 308 bp fragment in the first cycle and a 143 bp fragment in the second cycle of PCR [23].

### Phylogenetic analysis

Positive HAdV PCR products were purified using the QIAquick PCR purification kit (Qiagen, USA) and confirmed by sequencing (Sequencing unit, School of Medicine, University of Thessaly, Greece). The obtained nucleotide sequences were analyzed by BLAST program at the NIH website (NCBI, National Centre for Technology Control, NIH, USA), and were compared with each other as well with other published sequences. Multiple alignments were performed with the Clustal X program. The neighbour-joining method was applied for the phylogenetic tree analysis, the reliability of which was assessed by bootstrap resampling (1000 pseudoreplicates), using MEGA 4.0.2 program.

### Statistical analysis

Data were analyzed using IBM SPSS 22.0 (SPSS Inc., Chicago, IL, USA). Attack rate was calculated based on population data provided by the National Census of 2011 [24]. Data were analyzed using descriptive statistics whereas differences in demographic variables, between the years 2011 and 2012 were evaluated by Student’s *t*-test. Also comparisons age with sex were performed with Anova test. A *P* value lower than 0.05 was considered significant, for all statistical analyses. All values are expressed as mean (SD).

## Results

### Epidemiological investigation

**Fig. 1:**
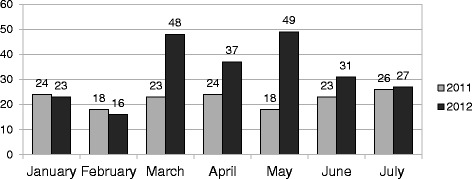
Number of conjunctivitis cases referred to the General University Hospital of Patras (weeks 1–30, years 2011 and 2012). The incidence of infectious conjunctivitis cases increased between March and June 2012 compared to the corresponding period in 2011 (*p* < 0.005, t-test)

**Fig. 2:**
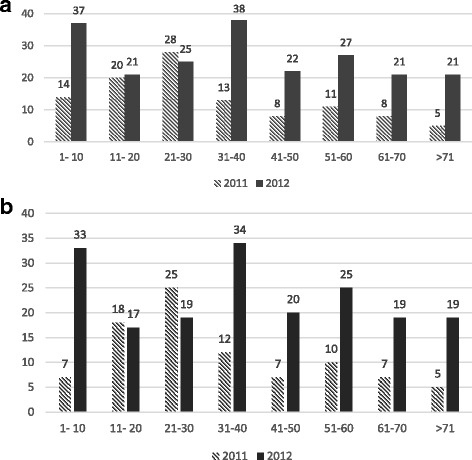
**a** and **b**: Figures show the total and virological conjunctivitis cases by age. **a** Total conjunctivitis cases by age **b**) Virological conjunctivitis cases by age

**Fig. 3:**
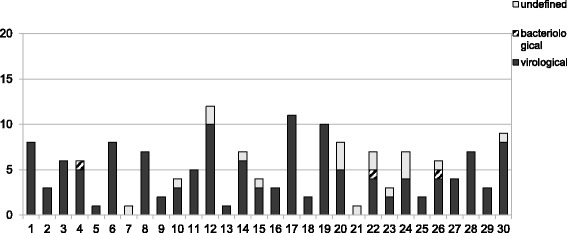
Number of infectious conjunctivitis cases per week in General University Hospital of Patras, during the period w1 to w30

A total of 231 patients (47.2% male and 52.8% female, aged 1 to 95 years old) suffering from conjunctivitis were referred to the GUHP from January 1 till July 31 (weeks 1–30). The diagnosis of the type of conjunctivitis was clinical. Allergic conjunctivitis cases were excluded from the study. Based on the clinical features of the 231 cases, 205 cases (88.7%) were of viral origin (46.3% male and 53.7% female) and only 4 cases were referred as bacterial conjunctivitis (50% male and 50% female). A total of 22 cases were undefined conjunctivitis. Between February 27, 2012 to June 17, 2012 (weeks 9–24), a total of 156 cases have been recorded (38.5% male and 61.5% female). Of these cases, 134 referred as viral conjunctivitis (37.3% male and 62.7% female), 19 as undefined conjunctivitis and 3 as bacterial conjunctivitis (66.7% male and 33.3% female). In 47.9% of the 134 cases of viral conjunctivitis the insult was bilateral. Eyelid oedema was evident in 97.9%, subconjunctival hemorrhages in 72.9%, pseudomembranes in 12.5% and in the 54.2% of the cases there was an ipsilateral pre-auricular lymphadenopathy. Keratoconjunctivitis occurred in 50% of the cases and in 29.2% of the keratoconjunctivis cases subepithelial corneal infiltrates were evident. When asked, 29.2% of the patients reported possible connection with another viral conjunctivitis case in their social environment. The incidence of infectious conjunctivitis cases in 2012 was like that of January, February and July 2011 and increased between March and June 2011 (*p* < 0.05, t-test), as it is shown in Fig. 1. Figure 2a and b show the total conjuctivitis and virological conjuctivitis cases stratified by age groups. Figure 3, shows the weekly number of infectious conjunctivitis cases admitted to the hospital between January 1st and July 31th 2012 (weeks 1–30). The epidemic curve is based on week of reference to the hospital and shows a sharp increase of conjunctivitis cases during March, as well as high occurrence of cases observed during May. The suspected excess cases started in March and peaked in May. There was no statistically significant association between the age and the gender over the course of the outbreak (*p* = 0.406, anova).

As it concerned the environmental data, there was an increased mean precipitation and humidity in months March to June in 2012 compared with the precipitation and humidity in the corresponding period in 2011. There was no difference in mean temperature between the two period times.

### Molecular analysis

From the period of the excess cases (2 February till 17 June, weeks 9–24), 48 conjunctival swab samples were collected. Swab specimens were obtained from 48 in-and out-patients with a clinical presentation compatible with viral conjunctivitis referred to the GUHP (36.4% of total viral cases). PCR was undertaken for all the 48 swab specimens. Forty samples (83.3%) were PCR positive and eight were negative. HAdV 17 in 29 samples (72.5%) and HAdV 54 in 5 samples (12.5%) were isolated and identified by PCR and sequencing. For six positive samples (15%) the types were not determined. Sequence BLAST search, indicated that these outbreak isolates were adenoviruses type D and probably strain 17, presenting a mean nucleotide identity of 94.8% (86–97%) to the adenovirus strain 17′ H30 (Genbank: EF195772.1 human adenovirus 17 strain 17′ H30). Additionally, five isolates were typed as AdV type D, probably strain 54, presenting a mean nucleotide identify of 94.8% (86–97%) to adenovirus 54 (Genbank: AB448770.2 human adenovirus 54). There was no statistically significant association of patients’ habits or routes of exposure to the identified viral types (probably due to the small size of sample).

### Phylogenetic analysis

**Fig. 4:**
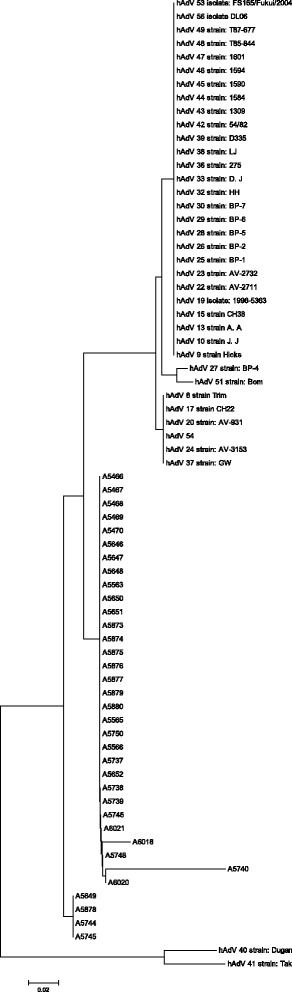
Dendrogram tree constructed to represent phylogenetic relationships among 34 HAdV strains, (abbreviated as Axxxx) isolated from Patras’ eye swab samples, along with 35 HAdV-D reference sequences. Two HAdV-F strains (HAdV40, Dukan, and HAdV41, Tak) [were used as out-groups for the analysis, and form a distinct clade to all human strains, as expected

Figure 4 shows a dendrogram constructed to represent phylogenetic relationships among 34 HAdV strains (abbreviated as Axxxx) which were isolated from Patras’ eye swab samples, along with 35 HAdV-D reference sequences. Two HAdV-F strains (HAdV40, Dukan, and HAdV41, Tak) were used as out-groups for the analysis, and form a distinct clade to all human strains, as expected.

## Discussion

Based upon our knowledge, this is the first study describing an HAdV outbreak in Greece, both epidemiologically and phylogenetically. This outbreak is remarkable because of the significant number of affected subjects, the wide geographical area involved, and the investigation performed, which is not common in Greece.

Laboratory confirmation of the diagnosis can help physicians to rapidly undertake suitable hygienic measures, and determine the epidemiological significance of the infection. Nucleic acid amplification techniques are the diagnostic approach of choice, because of their high sensitivity, high specificity, and rapidity [25]. As there is neither an effective treatment for EC nor a vaccine against it, hygienic measures are of paramount importance in preventing the spread of infection [25].

The epidemic curve presented a high occurrence of adenoviral conjunctivitis cases between March and May. The number of cases declined slowly in June and the epidemic finally stopped in July. This epidemic curve suggested a common source of contamination, but the initial source was not identified in this study. The outbreak affected all age groups (average 37.7 years). Age peaks occur in the 1–10 and 31–40 age groups. Gender was not associated with adenoviral types in adenoviral conjunctivitis in our study population, which is consistent with the finding reported previously [26, 27]. There was a possible correlation of increased precipitation and humidity in months in 2012 (compared with 2011) with the increased number of cases of conjuctivitis. There was no statistically significant association of patients’ habits or routes of exposure to the identified viral types (probably due to the small size of sample).

In the present study, although only a low number of samples was analyzed, the high prevalence of HadV D and probably type 17 between March and June 2012, suggests that this virus could have been the etiologic agent of excess conjunctivitis cases in Patras. Additionally, HAdV54 was isolated from some eye swab specimens, which has been determined as an emerging type. EC is typically reported to be caused by adenovirus types 8, 9, and 37, which frequently cause nosocomial outbreaks. Other adenovirus types have rarely been associated with EC29. This is the first description of an excess conjunctivitis cases caused possibly by adenovirus type 17.

Adenovirus 17 was isolated from conjunctival scrapings in 1955 and designated Ch.22. This adenovirus was grouped into species HAdV-D and confirmed [28]. On the other hand, adenovirus 54 was mainly associated with outbreaks of conjunctivitis infection in Japan. Also, the origin and route of transmission of HAdV54 are unknown. It is also possible that this virus is circulating in humans with asymptomatic infections. The novel type HAdV54 should, therefore, be monitored worldwide as an emerging HAdV strain. HAdV54 has the potential to cause an outbreak of EC in institutions and communities. In the early stage of the disease, it is frequently misdiagnosed as acute allergic conjunctivitis. Therefore, an early detection of HAdV54 in a clinical setting is required. A molecular test for adenovirus should be performed in ophthalmology clinics to differentiate between the early stage of HAdV54 EKC and acute allergic conjunctivitis. Laboratory confirmation using PCR is also recommended for the early detection of an HAdV54 outbreak [29, 30].

Frantzidou et al. [18] conducted a study to investigate the molecular epidemiology of adenovirus strains isolated from patients with ocular disease in Thessaloniki, Northern Greece, between 1998 and 2002. HAdV strains belonging to types 2, 3, 4, 8, and 15, were isolated.

Also, Moustaka et al. [19] conducted a study to investigate the genotypes and variant types of adenoviruses causing keratoconjunctivitis in Athens, Greece, during a 4 months’ period (from January until April 2010). All positive samples showed types HAdV8, HAdV19, and HAdV37. HAdV8 was found to be the predominant type.

In Greece, there is no reporting surveillance system for conjunctivitis outbreaks, and therefore many conjunctivitis cases or cases that occurred during the outbreak may not have been recorded. This could be a fact mainly for mild cases or cases occurring in rural areas. Several patients probably contacted private physicians, or rural Health Centers existing in the studied area. Such patients were not recorded as part of the cases of the studied outbreak.

Sequence BLAST search confirmed that the outbreak isolates belonged to adenovirus type 17 and 54. The use of short sequences has been successful in establishing diagnoses of adenoviral infection, but it becomes problematic for the classification or phylogenetic analysis. Molecular typing of at least one of the two hypervariable loops, which constitute the neutralization epitope of the hexon protein, could have been more accurate, compared to the sequencing of the conserved parts of the hexon. Recently, HAdV53, HAdV54, and HAdV56 have been identified as a cause of conjunctivitis, and these viruses are closely related to HAdV8, HAdV19, and HAdV37. Interestingly, HAdV54 has not yet found as a significant conjunctivitis agent in Europe. Moreover, HAdV17 has not yet been described as a conjunctivitis agent.

Further investigations supported by sequencing of the fiber knob and the penton base hypervariable loops, or by complete genomic sequencing are needed to elucidate the molecular typing of the isolated strains.

Due to lack of a surveillance system for acute conjunctivitis in Greece, the accomplishment of the study proved very difficult. The duration is likely due to many cases related to person-to-person contamination. Also, the present epidemiological study confirmed the necessity of the development of a surveillance system for acute conjunctivitis outbreaks in Greece.

## Conclusion

The epidemiological as well as molecular investigation on HAdV ocular infections is rather absent in Greece, which has no surveillance system for conjunctivitis. There is a significant need for a surveillance system for acute infectious conjunctivitis outbreaks as it will contribute significantly in the protection as well as health promotion of eye infections.

## Abbreviations

BLAST: Basic Local Alignment Search Tool
EC: Epidemic conjunctivitis
HAdVs: Human Adenoviruses
NCBI: National Centre for Technology Control
NIH: National Institutes of Health
UHP: University Hospital of Patras
